# The Impact of Obesity on the Health of the Older Population: A Cross-Sectional Study on the Relationship between Health-Related Quality of Life and Body Mass Index across Different Age Groups

**DOI:** 10.3390/nu16010051

**Published:** 2023-12-22

**Authors:** Patrick Reinbacher, Alexander Draschl, Maria Anna Smolle, Andrzej Hecker, Franz Gaderer, Kay-Bernd Lanner, Paul Ruckenstuhl, Patrick Sadoghi, Andreas Leithner, Stefan Nehrer, Thomas Klestil, Kevin Brunnader, Gerwin A. Bernhardt

**Affiliations:** 1 Department of Orthopaedics and Trauma, Medical University of Graz, 8036 Graz, Austria; patrick.reinbacher@medunigraz.at (P.R.); paul.ruckenstuhl@medunigraz.at (P.R.); patrick.sadoghi@medunigraz.at (P.S.); andreas.leithner@medunigraz.at (A.L.); kevin.brunnader@gmail.com (K.B.); wintschgerl@gmail.com (G.A.B.); 2 Faculty Health & Medicine, University for Continuing Education, 3500 Krems, Austria; franz.gaderer@kepleruniklinikum.at (F.G.); kb@lanner-md.at (K.-B.L.);; 3 Division of Plastic, Aesthetic and Reconstructive Surgery, Department of Surgery, Medical University of Graz, 8036 Graz, Austria; 4 COREMED-Centre for Regenerative and Precision Medicine, Joanneum Research Forschungsgesellschaft mbH, 8010 Graz, Austria; 5 Department of Orthopaedics and Trauma, Johannes Kepler University Linz, 4040 Linz, Austria; 6 Department of Orthopaedics and Trauma, Kantonsspital St. Gallen, 9007 St. Gallen, Switzerland; 7 Department of Orthopaedics and Traumatology, University Hospital Krems, Karl Landsteiner University of Health Sciences, 3500 Krems, Austria; 8 Department of Orthopaedics and Trauma, Landesklinikum Baden-Mödling, 2340 Mödling, Austria

**Keywords:** body mass index, overweight, obesity, health-related quality of life, SF-36

## Abstract

Obesity is strongly associated with mortality and morbidity, but there is a lack of data on its impact on health-related quality of life (HRQoL) across different age groups. Therefore, this study’s objective was to determine the association between body mass index (BMI) and HRQoL in the Austrian adult population based on age groups using the 36-Item Short Form (SF-36) survey. Methods: The SF-36 survey was sent to 500 randomly assigned Austrian adults (response rate: 80.6%). This study assessed HRQoL subscale and component scores based on gender, level of education, smoking status, and alcohol consumption in 403 participants. Results: Increasing BMI is associated with a negative impact on all domains of physical health and social function. The study uncovered substantial variations in the impact of increasing BMI on HRQoL across different age groups, with a pronounced effect observed in the physical components, particularly among individuals aged 65–74. Conclusions: BMI is negatively associated with the physical aspects of HRQoL and social function, affecting various adult age groups differently. Consequently, our results emphasize assessing different age groups and possible influencing factors on HRQoL, such as BMI, for further optimization in designing prevention programs against obesity.

## 1. Introduction

The global prevalence of overweight and obesity (OO) has significantly increased in recent decades. Most of the world’s population lives in countries where OO kills more people than underweight [1]. According to the World Health Organization (WHO), over 1.9 billion adults worldwide were overweight and 650 million were obese in 2016 [1]. Reflecting these numbers, 39% of adults aged 18 years or older were overweight, and 13% were obese [1].

OO and its associated complications can have a detrimental impact on health-related quality of life (HRQoL) [2]. Numerous studies have highlighted the association of OO with various adverse medical conditions, including psychological problems, cardiovascular disease, diabetes, and cancer [3,4,5,6,7,8,9,10,11,12,13,14,15,16]. In terms of depression onset in adulthood, maintaining a persistently high BMI from childhood to adulthood emerges as a significant risk factor. Even individuals with a normal BMI during childhood are more likely to suffer from depression if their BMI gradually increases over time [17]. Moreover, it has been demonstrated that the beneficial effects of dietary fibers on the intestines may be diminished in obese children with pre-existing insulin resistance [18]. Furthermore, individuals classified as obese based on BMI classification face an increased risk of postoperative complications, as evident in hip arthroplasty [19,20]. In such cases, they are more susceptible to deep infections and femoral stem subsidence compared to normal-weight individuals. In addition to its negative impact on HRQoL, obesity is associated with higher costs for the health care system, higher care and maintenance, and shorter life expectancy [21,22,23].

HRQoL is a multimodal concept and an important patient-reported outcome measure (PROM) that assesses the multidimensional nature of subjective well-being, including physical, mental, emotional, and social functioning [24]. In general, it focuses on the awareness of one’s state of health [25,26]. The 36-Item Short Form (SF-36) health survey has become one of the most widely used PROM to assess HRQoL, determining physical and mental components [24,27]. According to the existing literature, obese and overweight patients have worse HRQoL scores than normal-weight patients [28,29]. Previous studies have demonstrated a more negative impact of obesity on the physical domains of HRQoL than on the mental domains [28,30,31,32,33,34]. However, a meta-analysis [28] suggests that increasing BMI to a certain degree positively affects mental health. The relationship between BMI and HRQoL seems to be a complex interplay of several factors, including the level of education and various other determinants. The variability in results among studies highlights the complexity of this relationship [35]. Although the negative association between obesity and HRQoL is well established, only a few studies have investigated this relationship, and the existing literature is mainly limited to specific subpopulations [36,37,38,39,40].

Furthermore, there is a lack of data on whether particular groups in the general population are at greater risk for lower HRQoL scores [28]. Given the anticipated rise in the prevalence of OO, a better understanding of its various consequences, including its impact on HRQoL, can assist policymakers and healthcare professionals in developing preventive measures [25,26,41,42]. Due to the multifaceted nature of the effects of BMI on HRQoL, the present study aims to conduct a more comprehensive investigation by analyzing the relationship between BMI and HRQoL across different age groups. Additionally, this study seeks to provide valuable HRQoL data for a representative population of Austrian adults while considering additional factors such as gender, age, smoking status, and alcohol consumption, which have not been consistently reported in previous studies. Through these efforts, this study will contribute to the existing literature and provide novel insights into the complex relationship between BMI and HRQoL in the general population.

Our study aims to present the importance of a healthy lifestyle in maintaining a normal weight and raise public awareness on this matter. The diseases and consequences associated with obesity have a significant impact not only on our healthcare providers but also on the general population, and not just in financial terms.

## 2. Materials and Methods

The study was conducted in accordance with the Declaration of Helsinki and approved by the Institutional Review Board of the Medical University of Graz (protocol code 14-143 ex 03/04).

### 2.1. Study Design and Data Sources

The SF-36 survey was sent to 500 randomly assigned Austrian adults. For the purposes of participant recruitment in our study, we used the Austrian phone directory as a sampling frame and, through a two-stage stratified randomization process, identified a cohort of 500 adult individuals. To ensure a proportionate representation of the various geographical regions, the phone book was first separated into strata based on the nine federal states of Austria. A predefined number of pages were chosen at random from each stratum using a systematic random sampling technique via Microsoft Excel (Microsoft Corporation (2023). Microsoft Excel (Version 16.78). Redmond, WA, USA). To guarantee that every listing had an equal chance of being chosen, a uniform number of listings were chosen at random from the pages inside each stratum in the second stage using the same randomization technique. We then contacted these individuals via telephone, and sent them the SF-36 questionnaire via post. Participants were informed about the purpose of the study, the confidentiality of their responses, and their right to withdraw from the study at any time.

### 2.2. Health-Related Quality of Life and Subjective Outcome Measurement

Besides the two main dimensions of health, the physical (PCS) and mental (MCS) component scores, the SF-36 survey is composed of eight subscales that portray various domains of HRQoL, including physical function (PF), role physical (RP), bodily pain (BP), general health perception (GH), vitality (VT), social function (SF), role emotional (RE) and mental health (MH) [24,43]. The SF-36 is a patient-reported questionnaire used to survey health status (HS) and HRQoL, enabling individuals to describe their health status subjectively. On its 100-point scale, high scores equate to good health, and low scores correlate to poor health.

### 2.3. Body Mass Index (BMI)

The body mass index is a simple and helpful score to compare study participants’ weight. The BMI is defined as a person’s weight in kilograms divided by the square of their height in meters [1]. According to the WHO, a person’s BMI of 25 kg/m^2^ or greater is classified as overweight, and a BMI of 30 kg/m^2^ or greater is classified as obese [44]. Obesity was further divided into class I, defined as 30.0–34.9 kg/m^2^, class II, defined as 35.0–39.9 kg/m^2^, and class III, defined as ≥40 kg/m^2^ [13].

### 2.4. Statistical Analysis

All statistical analyses were performed with Stata Version 15.1 (StataCorp, College Station, TX, USA). Means and medians are provided with standard deviations and interquartile ranges, respectively. For the comparison of continuous variables and binary parameters, *t*-tests were performed. One-way analysis of variance (ANOVA) was performed to assess differences between continuous variables. Two continuous variables were compared with linear regression analysis. A *p*-value of less than 0.05 indicated a statistically significant difference.

The eight subscale scores of the SF-36 survey (PF, RP, BP, GH, VT, SF, RE, and MH) and the dichotomized component scores (PCS and MCS), sets of scores of which were described and established in previously conducted studies [24,45,46], were considered for statistical analysis. In addition, to assess the influence of different aspects on HRQoL, the measurements were analyzed concerning gender, age, education level, BMI, smoking status, and alcohol consumption.

## 3. Results

Our sample consisted of a total of 403 participants. The response rate was 80.6% (403/500), with varying response rates for each category, as displayed in Table 1 and Table 2. The demographic data and characteristics of the study population are presented in Table 1. Most participants (35.2%) were classified as class III obese, followed by normal-weight individuals (35.0%) and overweight individuals (23.3%). The gender distribution was roughly equal, with females accounting for 51.1% of the population. The age distribution was mostly even, except for a small proportion of participants who were 85 years or older (2.2%). In terms of education, the highest level achieved by participants was predominantly vocational school (34.6%), followed by A-levels (28.6%), a university diploma (25.2%), primary school (9.0%), and an apprenticeship (2.6%). While 78.0% of participants were non-smokers, 98.1% of individuals reported consuming alcohol.

Table 2 displays the characteristics based on BMI classification [13]. The study population exhibited a diverse distribution across different BMI classifications. The majority of participants fell into the class III obese category (35.2%), followed by normal-weight (34.9%), overweight (23.3%), class I obese (5.2%), class II obese (0.74%), and underweight (0.50%) individuals. The gender distribution was comparable across all BMI classification groups, except for the underweight group (female: 100.0%) and obesity class II group (female: 0.0%). In terms of education, vocational school was the most common highest level achieved among overweight (40.4%) and class I obese people (47.6%), while most class III obese individuals (85.7%) reported having a university diploma. Regarding smoking status, non-smokers were more prevalent among normal-weight (76.6%), overweight (78.3%), class I obese (90.5%), and class III obese (100.0%) participants, while the underweight (50.0%) and obesity class II (33.3%) groups each had one non-smoker. Conversely, most participants in all BMI groups reported consuming alcohol.

### 3.1. Health-Related Quality of Life and Body Mass Index

We performed regression analyses to assess the relationship between BMI and HRQoL parameters. As presented in Table 3, BMI shows a statistically significant negative correlation with PF (*p* < 0.001), RP (*p* < 0.001), BP (*p* < 0.001), GH (*p* < 0.001), SF (*p* = 0.027), and PCS (*p* < 0.001). On the other hand, a positive correlation was observed between BMI and MCS (*p* < 0.001).

### 3.2. Health-Related Quality of Life and Body Mass Index Based on Age Groups

Furthermore, we performed regression analyses to obtain the relationship between BMI and the HRQoL parameters based on the age groups. No statistically significant correlations between BMI and the subscale scores were observed for individuals aged < 35 years. We found that participants aged 35–44 years have a significant negative correlation between BMI and RP (*p* = 0.008), BP (*p* < 0.001), as well as PCS (*p* = 0.004). A negative correlation between BMI and BP (*p* = 0.004), as well as BMI and MH (*p* = 0.010), was observed in 45 to 54-year-old individuals. No statistically significant correlations were found in individuals aged 55–64 years. Negative correlations were found in 65 to 74-year-old participants in case of increased BMI and PF (*p* < 0.001), RP (*p* = 0.001), BP (*p* = 0.001), GH (*p* < 0.001), VT (*p* = 0.003), SF (*p* < 0.001), RE (*p* = 0.007), MH (*p* = 0.014), as well as PCS (*p* < 0.001). Individuals over 74 years showed no statistically significant correlations between BMI and the HRQoL parameters (Supplementary Table S1).

### 3.3. Health-Related Quality of Life and Gender

T-tests were performed to assess differences in HRQoL scores between genders. Although male participants reported better overall HRQoL scores, except for MCS, the differences were not statistically significant (Supplementary Table S2).

### 3.4. Health-Related Quality of Life and Age Groups

Health-related quality of life (HRQoL) parameters across different age groups were compared (Supplementary Table S8). Statistically significant differences were mainly identified in the physical health domain and its subscales. Participants aged 65–74 reported significantly worse results in all physical health parameters, but better MCS (*p* < 0.001) than participants aged <35. Furthermore, individuals aged ≥75 years showed significantly inferior results to all younger age groups regarding PF, RP, and PCS. Regarding mental health, we could only identify statistically significant differences between the age groups in the MCS; here, its subscale of RE individuals aged ≥75 showed significantly inferior results than individuals aged 55–64 (*p* = 0.003). Apart from that, the other corresponding subscales (VT, SF, MH) showed no significant differences.

### 3.5. Health-Related Quality of Life and Educational Level

This study categorized educational levels into five groups based on an individual’s highest level of education: (1) primary school, (2) apprenticeship, (3) vocational school, (4) A-levels and (5) university diploma. T-tests were performed to compare HRQoL parameters between men and women within each educational level, and no statistically significant differences were found between genders (Supplementary Table S4).

HRQoL parameters based on educational level were compared (Supplementary Table S9). The university diploma group had the highest PF scores, but the difference was insignificant compared to the A-levels group (*p* = 0.482). The A-levels group only reported significantly better PF scores than the primary school group (*p* < 0.001). RP scores were significantly better in the A-levels group compared to the primary school (*p* < 0.001) and vocational school (*p* = 0.024) groups. Higher BP scores were observed in the university diploma group compared to the primary school (*p* < 0.001) and vocational school (*p* < 0.001) graduates. Likewise, the A-levels group had significantly better BP scores than primary school (*p* < 0.001) and vocational school (*p* = 0.001) graduates. Vocational school graduates only had superior results to the primary school group (*p* = 0.011). GH was significantly better in the A-levels group than in the primary school group (*p* = 0.014). Individuals with a university diploma had better results than primary school (*p* = 0.001) and vocational school (*p* = 0.001) graduates for GH. A-levels and university graduates had significantly better VT (*p* = 0.017, *p* = 0.021, respectively) and SF (*p* = 0.018, *p* = 0.029, respectively) scores than primary school graduates. The vocational school, A-levels, and university diploma groups had significantly better RE scores than primary school graduates (*p* = 0.037, *p* = 0.002, *p* = 0.001, respectively). There was no significant difference in MH scores between the groups. Similarly to the RP scores, A-levels graduates had significantly better PCS scores than primary school (*p* < 0.001) and vocational school (*p* = 0.007) graduates, and university diploma graduates had significantly better PCS scores than primary school (*p* < 0.001) and vocational school (*p* < 0.001) graduates. Moreover, individuals with a university degree had significantly worse MCS scores than primary school (*p* = 0.023) and vocational school (*p* = 0.006) graduates.

### 3.6. Health-Related Quality of Life and Educational Level Based on Age Groups

Among participants aged less than 35 years, no statistically significant differences were observed in HRQoL parameters when considering different educational levels. In the 35–44 years age group, PF scores were significantly higher among vocational school (*p* < 0.001), A-level (*p* < 0.001), and university diploma (*p* < 0.001) graduates than primary school graduates. Furthermore, RP scores were significantly higher among university diploma graduates than primary school graduates (*p* = 0.030). In the 45–54 years age group, university diploma graduates reported better RP (*p* = 0.017), BP (*p* = 0.020), and RE (*p* = 0.022) than A-level graduates. Participants who were 55–64 years of age holding a university degree had better PF (*p* = 0.030), RP (*p* = 0.028), GH (*p* = 0.003), and PCS (*p* = 0.008) scores than vocational school graduates, and A-level graduates reported a significantly better PCS than the vocational school group (*p* = 0.020). For individuals aged 65 to 74 years, A-level graduates had superior results in BP (*p* = 0.032) and RE (*p* = 0.027) than primary school graduates, and university degree holders had better results in terms of RP (*p* = 0.019), BP (*p* = 0.042), RE (*p* = 0.032), and PCS (*p* = 0.017) than the primary school group. The ≥75 years age group only revealed statistically significant differences in BP, with A-level graduates reporting better results than the primary school (*p* = 0.0014) and vocational school (*p* = 0.006) groups, and participants with a university diploma reported better results than A-level graduates (*p* = 0.030) (Supplementary Tables S5.1–S5.7).

### 3.7. Health-Related Quality of Life and Smoking Status

A *t*-test evaluating differences in HRQoL parameters between smokers and non-smokers revealed no statistically significant differences (Supplementary Table S6). We additionally obtained information on differences in the parameters between smokers and non-smokers, performing *t*-tests based on the age groups (Supplementary Table S7). No statistically significant differences between smokers and non-smokers could be observed in participants aged under 35 years. However, non-smokers aged 35–44 years reported significantly better MCS (*p* = 0.019), MH (*p* < 0.001), RE (*p* < 0.001), SF (*p* < 0.008), VT (*p* = 0.029), and GH (*p* = 0.002). Non-smokers aged 45–54 only had significantly better results regarding RE (*p* = 0.044), while no significant differences were observed in the older age groups (55 years and above).

## 4. Discussion

The main finding of this study includes the negative correlation between BMI and physical health (PF, RP, BP, GH, PCS), as well as SF. We could confirm low physical health scores of HRQoL in our analyses, which are congruent with the published literature [30,47,48,49]. However, we observed a non-significant positive correlation between BMI and mental health regarding the MCS in all age groups. Furthermore, our results show an impact of increasing BMI on HRQoL across different age groups, with a pronounced effect observed in the physical components, particularly among individuals aged 65–74. Therefore, our data indicate that the interpretation of the impact of BMI on HRQoL should be made with the different age groups in mind. In addition, as there is a lack of data comparing the influence of BMI across different age groups, the current study shows one of its strengths in providing valuable reference data for future studies.

Among individuals aged 35–44, increased BMI was associated with lower scores in all HRQoL parameters, except for MCS, where the positive correlation with BMI was not statistically significant. The tendency for higher MCS with increasing BMI is consistent with the results of a meta-analysis, which found that overweight patients had higher mental health scores than normal-weight individuals, whereas those with class III obesity (BMI of ≥40 Kg/m^2^) had significantly lower mental health scores [49]. This phenomenon can be explained by the so-called “jolly-fat” hypothesis, which suggests that obesity may have a protective effect on developing depression [50,51]. Conversely, our study revealed a significant negative correlation between BMI and RP (*p* = 0.008), BP (*p* < 0.001), and PCS (*p* = 0.004), indicating that physical domains of HRQoL are primarily affected by elevated BMI in this age group. These individuals may experience negative impacts on RP, BP, and overall physical health due to the strain that excess weight puts on the body, potentially leading to the development of joint pain and joint disease, which can negatively impact physical functioning [6,31,52]. According to a large survey study, osteoarthritis is one of the conditions that can significantly reduce physical functioning, resulting in a decrease in the physical component of HRQoL and a reduction in the overall score of HRQoL [53]. Even after total joint replacement, for example, after total hip arthroplasty, a high BMI is considered a risk factor for subsidence, which may lead to early revision surgery [20].

Individuals aged 45–54 years reported results similar to those aged 35–44, except for non-significant negative correlations between BMI and RP (*p* = 0.277) and PCS (*p* = 0.233) and a significant negative correlation between BMI and MH (*p* = 0.010). Furthermore, unlike in the younger age group (35–44 years), a non-significant positive correlation was observed between BMI and SF (*p* = 0.971) in individuals aged 45–54. These results can again be explained by the fact that age-related changes, such as the development of osteoarthritis and the increasing risk of comorbidities, can contribute to lower physical health [6,31,52]. Furthermore, older individuals may have a more developed coping mechanism for dealing with physical ailments [54,55,56], resulting in a less negative impact on RP and PCS. The age group of 45–54 years showed a significant negative association between BMI and MH, while such an association was not observed in the younger age group of 35–44 years. Our findings suggest that age-related psychological changes, such as cognitive decline and symptoms of depression, could potentially account for the observed difference in the relationship between BMI and MH between these two age groups [57,58,59]. Alternatively, older individuals may be more sensitive to changes in BMI due to age-related physiological changes, such as an increase in body fat and a decrease in lean muscle mass [60]. On the other hand, the non-significant positive correlation between BMI and the PF subscale score in individuals aged 45–54 years, unlike in the younger age group, may be attributable to age-related physiological changes and degeneration, which can result in diminished physical functioning irrespective of changes in BMI, as previously noted [60].

In this study, participants aged 55–64 demonstrated insignificant results in all HRQoL parameters, except for the MCS, while individuals aged 65–74 exhibited the poorest results regarding the negative correlation between BMI and HRQoL parameters. Despite similar correlations in both age groups, those aged 65–74 years showed significantly negative correlations (PF: *p* < 0.001; RP: *p* = 0.001; BP: *p* = 0.001; GH: *p* < 0.001; VT: *p* = 0.003; SF: *p* < 0.001; RE: *p* = 0.007; MH: *p* = 0.014; PCS: *p* < 0.001). This difference is likely the outcome of a complex interplay between various factors, including BMI, level of education, and numerous other determinants [35]. Moreover, HRQoL is a subjective concept, and prior research suggests a negative association between age and quality of life, primarily affecting the physical domains [29,55,58,61]. In other words, individuals may perceive their quality of life less positively as they age [57,58]. Furthermore, social roles may contribute to the observed results. Individuals aged 65–74 may be experiencing a period of life where they are more likely to confront the loss of their parents and the detrimental effects of retirement, which can negatively impact their quality of life [62,63,64]. However, our findings are consistent with those of Rambod et al. [65], who investigated the quality of life in the Iranian older population with a mean age of 68.0 years. The authors reported that overweight and obese individuals had a lower total quality of life than those with a normal weight [65]. Notably, a study of the Korean older population (over 60 years of age) [66] did not observe a negative impact of BMI on quality of life. In contrast, an investigation of Italian individuals aged 60 years or older [61] reported a negative impact of BMI on the physical subscales of quality of life, consistent with our findings. These discrepancies could also potentially result from methodological differences in assessing the quality of life. Nonetheless, the different findings reported in the literature indicate that geographic location also seems to have an equally crucial influence on the quality of life, adding to the multifactorial nature of this phenomenon. However, individuals older than 74 years exhibited a non-significant trend of a positive association between GH (*p* = 0.8284), MH (*p* = 0.528), and MCS (*p* = 0.435) and an increasing BMI. In contrast, all other parameters displayed a non-significant negative association with BMI.

Our study revealed that increasing BMI is negatively associated with physical aspects of HRQoL and social function, affecting various adult age groups differently (results Section 3.1). These findings align with previous studies demonstrating decreased physical and improved mental health with advancing age [55,67,68,69]. However, our data indicate that the members of a specific age group, namely 65–74-year-old individuals, are especially prone to a significantly lower HRQoL regarding physical health. The findings of Thomas et al. [55] further support the existence of a “paradox” wherein aging is linked to better mental health among older adults at the population level despite physical and cognitive decline. This phenomenon may be attributable to an active emotional reserve, which enables older adults to counter threats to their mental health, such as declining physical health, as well as changes in socioemotional selectivity in later life, resulting from a narrower horizon of life given the acknowledgment of mortality, and the alignment of aspirations with realities [55,70,71].

Several limitations to this study warrant acknowledgment. First, Hermann et al. reported that weight perception in adults often does not align with their actual weight, potentially leading to distorted findings [72]. Therefore, the accuracy of the participant’s reported weight information may impact the validity of our findings, as our results depend on the provision of the participants’ correct weight. Second, our findings rely on self-reported SF-36 data, which is a limitation attributable to the nature of the methodology. In addition, the stratification of the study population into different subgroups resulted in relatively small frequencies for each subgroup, limiting the generalizability of the current study’s findings. Moreover, this study focused exclusively on the Austrian population, with a two-stage stratified sampling approach implemented to mitigate potential biases regarding sample selection to yield a representative group, which restricts the applicability of the findings to a country-specific context. It is important to mention that our study was a cross-sectional study; therefore, it is not possible to draw conclusions regarding cause and effect. Sources of error include the “healthy volunteer problem”, where people who are already interested in their health and maintaining it contribute to surveys like the SF-36. In addition to the “healthy volunteer problem”, recall bias also contributes to a limitation in establishing a cause-and-effect relationship. Furthermore, it is important to acknowledge the impact of the measurement error problem caused by height loss as people age, which affects the assessment of the impact of BMI on health outcomes in older age groups [73]. Additionally, HRQoL is influenced by various factors beyond body weight, such as illnesses, injuries, education, and socioeconomic status [2,5,74]. Moreover, Karlsen et al. stated that the two summary scores (PCS and MCS) of the SF-36 survey have satisfactory validity in morbidly obese individuals, but that the validity of the eight subscale scores may be questionable [75]. Finally, we have to acknowledge the limitations of the two-stage randomization process. Therefore, caution must be applied in interpreting the data, as 35.24% of the current study’s participants can be classified as morbidly obese (obesity class III).

## 5. Conclusions

Our study highlights the significance of maintaining a normal BMI range to promote physical health. The results suggest age-related differences in the association between BMI and HRQoL. Therefore, clinicians should take note of these distinctions when evaluating and managing patients’ HRQoL. Specifically, individuals aged 65–74 may have markedly lower HRQoL scores due to increased BMI. The HRQoL results of different age groups can serve as a benchmark for future studies examining patient-specific HRQoL outcomes. Additionally, the impact of BMI on HRQoL may have more significant implications for patients than individuals within the normative population.

## Figures and Tables

**Table 1:** Patient demographics.

		*n*	%
BMI (*n* = 403)	Underweight	2	0.5%
	Normal	141	35.0%
	Overweight	94	23.3%
	Class I obesity	21	5.2%
	Class II obesity	3	0.7%
	Class III obesity	142	35.2%
Gender (*n* = 403)	Male	197	48.9%
	Female	206	51.1%
Age (*n* = 403)	<35 years	63	15.6%
	35–44 years	71	17.6%
	45–54 years	59	14.6%
	55–64 years	77	19.1%
	65–74 years	72	17.9%
	≥75 years	61	15.1%
Education ^a^ (*n* = 266)	Primary school	24	9.0%
	Apprenticeship	7	2.6%
	Vocational school	92	34.6%
	A-levels	76	28.6%
	University diploma	67	25.2%
Smoking status ^b^ (*n* = 264)	Non-smoker	206	78.0%
	Smoker	58	22.0%
Alcohol status ^c^ (*n* = 263)	Non-alcohol	5	1.9%
	Alcohol	258	98.1%

^a^ Missing data: 34.0% (*n* = 137) of study participants did not provide information on their level of highest education. ^b^ Missing data: 34.5% (*n* = 139) of study participants did not provide information on their smoking status. ^c^ Missing data: 34.7% (*n* = 140) of study participants did not provide information on their alcohol status. BMI, body mass index. Underweight, BMI < 18.5; normal weight, BMI 18.5 < 25; overweight, BMI 25 < 30; obesity class I, BMI 30 < 35; obesity class II, BMI 35 < 40; obesity class III, BMI ≥ 40. The educational level represents an individual’s highest level of education.

**Table 2:** Patient demographics based on BMI classification.

		Underweight (*n* = 2)		Normal Weight (*n* = 141)		Overweight (*n* = 94)		Obesity Class I (*n* = 21)		Obesity Class II (*n* = 3)		Obesity Class III (*n* = 142)	
		*n*	% *	*n*	% *	*n*	% *	*n*	% *	*n*	% *	*n*	% *
Gender (*n* = 403)													
	Male	0	0.0%	64	45.4%	50	53.2%	12	57.1%	3	100.0%	68	47.9%
	Female	2	100.0%	77	54.6%	44	46.8%	9	42.9%	0	0.0%	74	52.1%
Age (*n* = 403)													
	<35	0	0.0%	27	19.2%	4	4.3%	2	9.5%	0	0.0%	30	21.1%
	35–44	0	0.0%	24	17.0%	11	11.7%	1	4.8%	1	33.3%	34	23.9%
	45–54	0	0.0%	23	16.3%	9	9.6%	3	14.3%	0	0.0%	24	16.9%
	55–64	0	0.0%	22	15.6%	26	27.7%	8	38.1%	2	66.7%	19	13.4%
	65–74	0	0.0%	26	18.4%	22	23.4%	5	23.8%	0	0.0%	19	13.4%
	≥75	2	100.0%	19	13.5%	22	23.4%	2	9.5%	0	0.0%	16	11.2%
Education ^a^ (*n* = 266)													
	Primary school	0	0.0%	9	6.5%	11	11.7%	3	14.3%	1	33.3%	0	0.0%
	Apprenticeship	1	50.0%	4	2.9%	2	2.1%	0	0.0%	0	0.0%	0	0.0%
	Vocational school	0	0.0%	42	30.2%	38	40.4%	10	47.6%	1	33.3%	1	14.3%
	A-levels	0	0.0%	40	28.8%	31	33.0%	4	19.1%	1	33.3%	0	0.0%
	University diploma	1	50.0%	44	31.7%	12	12.8%	4	19.1%	0	0.0%	6	85.7%
Smoking status ^b^ (*n* = 264)													
	Non-smoker	1	50.0%	108	76.6%	72	78.3%	19	90.5%	1	33.3%	5	100.0%
	Smoker	1	50.0%	33	23.4%	20	21.7%	2	9.5%	2	66.7%	0	0.0%
Alcohol status ^c^ (*n* = 263)													
	Non-alcohol	0	0.0%	2	1.4%	3	3.3%	0	0.0%	0	0.0%	0	0.0%
	Alcohol	2	100.0%	137	98.6%	89	96.7%	21	100.0%	3	100.0%	6	100.0%

* Column percentage. ^a^ Missing data: 40.0% (*n* = 137) of study participants did not provide information on their level of highest education. ^b^ Missing data: 34.5% (*n* = 139) of study participants did not provide information on their smoking status. ^c^ Missing data: 34.7% (*n* = 140) of study participants did not provide information on their alcohol status. Underweight, BMI < 18.5; normal weight, BMI 18.5 < 25; overweight, BMI 25 < 30; obesity class I, BMI 30 < 35; obesity class II, BMI 35 < 40; obesity class III, BMI ≥ 40. The educational level represents an individual’s highest level of education.

**Table 3:** Linear regression model on health-related quality of life parameters and body mass index.

	Coef.	*p*-Value	95% CI	
PF				
	−1.37	**<0.001**	−2.10	−0.63
RP				
	−1.62	**<0.001**	−2.44	−0.79
BP				
	−2.44	**<0.001**	−3.30	−1.59
GH				
	−1.21	**<0.001**	−1.84	−0.58
VT				
	−0.53	0.121	−1.20	0.14
SF				
	−0.91	**0.027**	−1.71	−0.10
RE				
	−0.77	0.074	−1.61	0.07
MH				
	−0.44	0.190	−1.10	0.22
PCS				
	−0.82	**<0.001**	−1.15	−0.49
MCS				
	0.78	**<0.001**	0.35	1.20

BMI, body mass index. PF, RP, BP, GH, VT, SF, RE, MH, PCS, and MCS are abbreviations for the SF-36 survey’s subscale and component scores. PF, physical function subscale score; RP, role physical subscale score; BP, bodily pain subscale score; GH, general health subscale score; VT, vitality subscale score; SF, social function subscale score; RE, role emotional subscale score; MH, mental health subscale score; PCS, physical component score; MCS, mental component score; 95% CI, 95% confidence interval. The linear regression models show the association between BMI and the HRQoL (health-related quality of life) scores. Data are presented as unstandardized regression coefficients (Coef.) and 95% confidence intervals estimated by the linear regression model. Bold values indicate a significant association between BMI and the corresponding HRQoL parameter (*p* < 0.05).

## Data Availability

The datasets generated and/or analyzed during the current study are available from the corresponding author upon reasonable request.

## Supplementary Materials

The following supporting information can be downloaded at: https://www.mdpi.com/article/10.3390/nu16010051/s1, Tables S1–S9: HRQoL_Supplementary.
